# Supplementary material for: A dynamic over games drives selfish agents to win–win outcomes
Source: Proc Biol Sci. 2020 Dec 16;287(1941):20202630. doi: 10.1098/rspb.2020.2630 (PMC7779514; doi:10.1098/rspb.2020.2630)
Supplement: Figures S1 - S2 [file rspb20202630supp1.pdf]

Electronic Supplementary Material (ESM) for

A dynamic over games drives selfish agents to win-win outcomes

Seth Frey[1,*], Curtis Atkisson[2],
[1] Department of Communication, UC Davis, One Shields Ave, Davis, CA 95616
[2] Department of Anthropology, UC Davis, One Shields Ave, Davis, CA 95616

* Direct correspondence to Seth Frey: sethfrey@ucdavis.edu

SUPPORTING FIGURES

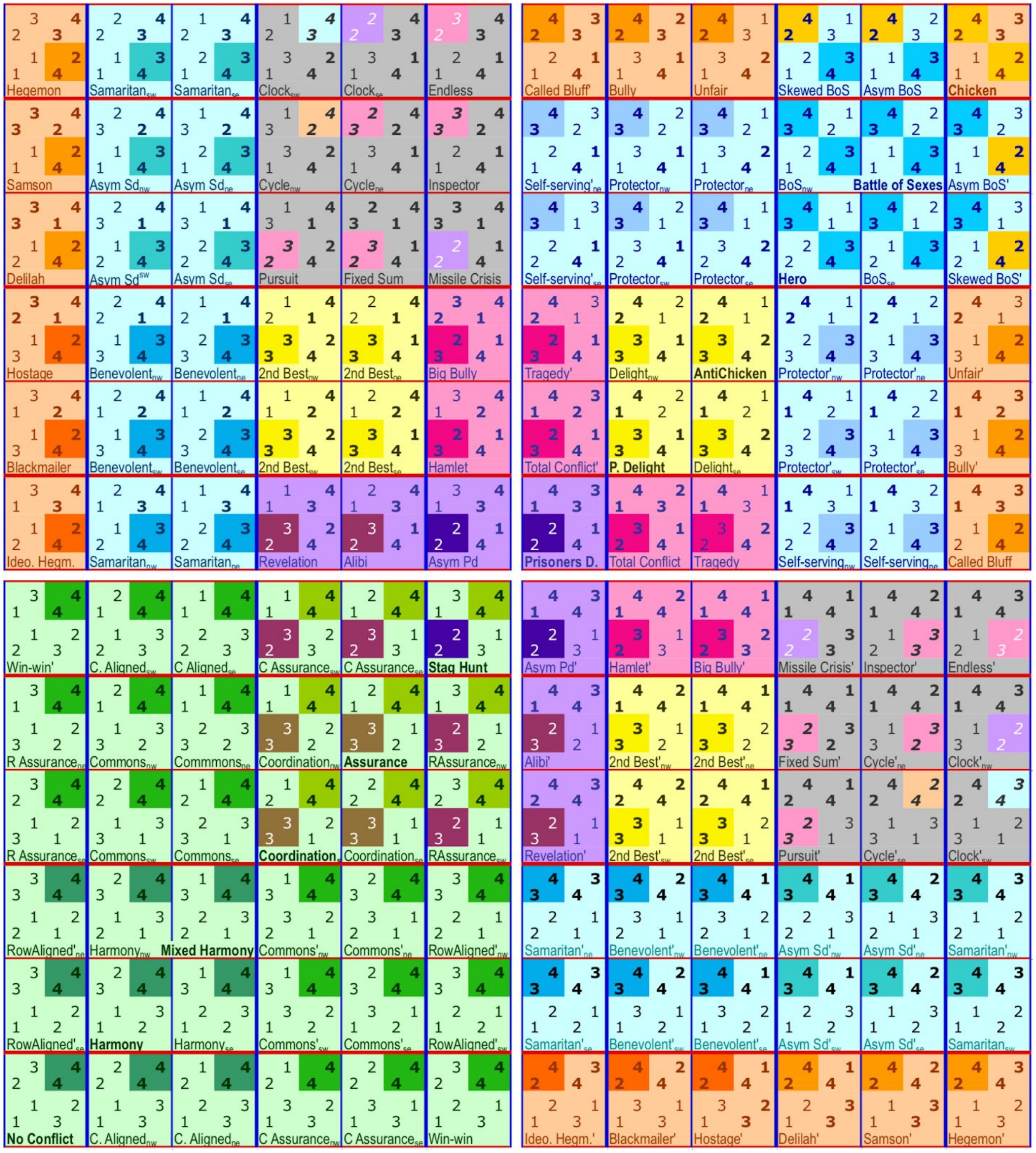

Figure S1. Richer visual representation of the topology of two-player, two-choice ordinal games.

This figure, an elaboration of Fig. 2a, uses different coloured games and different coloured game boundaries to illustrate different types of neighbour relations and game classes. Copied with permission from Bruns (2015).

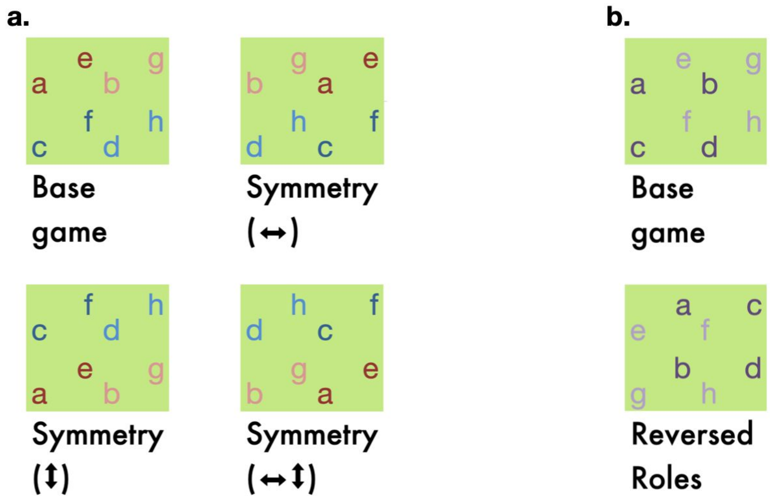

Figure S2. Symmetries of a game are subtracted from different count totals.

a. The four games in the above panel are identical in the sense that they differ only in the label each player's actions receive (Top vs Bottom; Left vs Right); strategically, the games are identical. After subtracting the symmetries, the 576 possible combinations of two players' rankings over four outcomes reduce to the 144 games of the two-player Topology of Games (Fig. S1). **b.** Another potential symmetry is one which presents the same game from the perspectives of each player. All games along the upper diagonal of Fig. 2a are symmetric in this sense. The main result, illustrated in Fig. 3b, shows the attractors of the self-interested dynamic with this symmetry subtracted: it shows only games that are attractors from the perspective of the "row" player, not the "column" player.
